# Fast Recall for Complex-Valued Hopfield Neural Networks with Projection Rules

**DOI:** 10.1155/2017/4894278

**Published:** 2017-05-03

**Authors:** Masaki Kobayashi

**Affiliations:** Mathematical Science Center, University of Yamanashi, Takeda 4-3-11, Kofu, Yamanashi 400-8511, Japan

## Abstract

Many models of neural networks have been extended to complex-valued neural networks. A complex-valued Hopfield neural network (CHNN) is a complex-valued version of a Hopfield neural network. Complex-valued neurons can represent multistates, and CHNNs are available for the storage of multilevel data, such as gray-scale images. The CHNNs are often trapped into the local minima, and their noise tolerance is low. Lee improved the noise tolerance of the CHNNs by detecting and exiting the local minima. In the present work, we propose a new recall algorithm that eliminates the local minima. We show that our proposed recall algorithm not only accelerated the recall but also improved the noise tolerance through computer simulations.

## 1. Introduction

In recent years, complex-valued neural networks have been studied and have been applied to various areas [1–5]. A complex-valued phasor neuron is a model of a complex-valued neuron and can represent phase information. Complex-valued self-organizing maps with phasor neurons have been applied to the visualization of landmines and moving targets [6–8]. Complex-valued Hopfield neural networks (CHNNs) are one of the most successful models of complex-valued neural networks [9]. Further extensions of CHNNs have also been studied [10–14]. In most CHNNs, the quantized version of phasor neurons, referred to as complex-valued multistate neurons, has been utilized. In the present paper, complex-valued multistate neurons are simply referred to as complex-valued neurons. Complex-valued neurons have often been utilized to represent multilevel information. For example, CHNNs have been applied to the storage of gray-scale images [15–17]. Given a stored pattern with noise, the CHNN cancels the noise and outputs the original pattern. In Hopfield neural networks, the storage capacity and noise tolerance have been important issues. To improve the storage capacity and noise tolerance, many learning algorithms for CHNNs have been studied [18, 19]. One such algorithm is the projection rule [20]. CHNNs have many local minima which decrease the noise tolerance [21].

CHNNs have two update modes, the asynchronous and synchronous modes. In the asynchronous mode, only one neuron can update at a time. In the synchronous mode, all the neurons simultaneously update and the CHNNs converge to a fixed point or to a cycle of length 2 [22]. Hopfield neural networks with the synchronous mode are also considered to be two-layered recurrent neural networks. In the present work, the synchronous mode is utilized. Lee proposed a recall algorithm for detecting and exiting the local minima and the cycles for the projection rule [23]. His recall algorithm improved the noise tolerance of the CHNNs. Since the local minima and the cycles are obviously the result of incorrect recall, it surely improves the noise tolerance to exit them.

In the present work, we propose a recall algorithm to accelerate the recall. Our proposed recall algorithm removes the autoconnections for updating and uses only the autoconnections for detecting the local minima and the cycles. Our proposed recall algorithm eliminates the local minima and the cycles. We performed computer simulations for the recall speed and the noise tolerance. As a result, we showed that our proposed recall algorithm not only accelerated the recall but also improved the noise tolerance.

The rest of this paper is organized as follows.  Section 2 introduces CHNNs and proposes a new recall algorithm. We show computer simulations in Section 3.  Section 4 discusses the simulation results, and Section 5 concludes this paper.

## 2. Complex-Valued Hopfield Neural Networks

### 2.1. Architecture of Complex-Valued Hopfield Neural Networks

In the present section, we introduce the complex-valued Hopfield neural networks (CHNNs). First, we describe the architecture of the CHNNs. The CHNNs consist of the complex-valued neurons and the connections between complex-valued neurons. The states of the neurons and the connection weights are represented by complex numbers. We denote the state of neuron *j* and the connection weight from neuron *k* to neuron *j* as *x*_*j*_ and *w*_*jk*_, respectively. Let *W* be the matrix whose (*j*, *k*) component is *w*_*jk*_. *W* is referred to as the connection matrix. The connection weights are required to satisfy the stability condition $w_{jk}={\bar{w}}_{kj}$, where $\bar{w}$ is the complex conjugate of *w*. Therefore, the connection matrix *W* is Hermitian. A complex-valued neuron receives the weighted sum input from all the neurons and updates the state using the activation function. Let *K* be an integer greater than 1. We denote *θ*_*K*_ = *π*/*K*. The activation function *f*(*I*) is defined as follows:

(1) fI=10≤arg⁡I<θKe2iθKθK≤arg⁡I<3θKe4iθK3θK≤argI<5θK⋮⋮e2K−1iθK2K−3θK≤arg⁡I<2K−1θK12K−1θK≤argI<2π.

*K* is the quantization level and *i* is the imaginary unit. Let *N* be the number of neurons. The weighted sum input *I*_*j*_ to neuron *j* is given as follows:

(2) $$\begin{array}{c} I_{j}=\overset{N}{\underset{k=1}{\sum }}w_{jk}x_{k}. \end{array}$$

**Figure 1:**
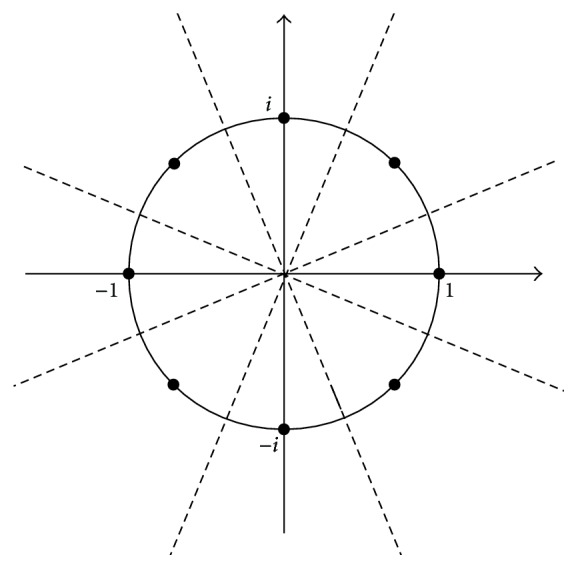
The activation function of complex-valued neurons in the case of *K* = 8. The dashed lines are decision boundaries.

Figure 1 shows the activation function in the case of *K* = 8.

**Figure 2:**
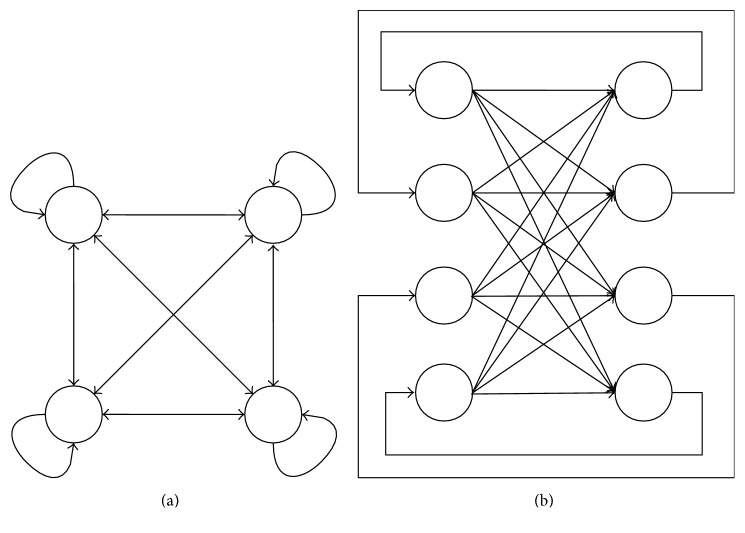
(a) A CHNN. (b) A CHNN represented with two layers. After the right layer updates, the state of the right layer is transferred to the left layer.

Next, we describe the recall process. There exist two updating modes, the synchronous and asynchronous modes. In the asynchronous mode, only one neuron can update at a time. If the connection weights satisfy the stability condition and the autoconnection *w*_*jj*_ of neuron *j* is positive for all *j*, the energy of the CHNN never increases and the CHNN always converges to a fixed point. In the synchronous mode, all the neurons update simultaneously and the CHNNs converge to a fixed point or to a cycle of length 2. In both modes, updating continues until the CHNNs converge to a fixed point or a cycle. In this work, we utilize the synchronous mode.  Figure 2(a) illustrates the network architecture of CHNN. In the synchronous mode, a CHNN can be also represented by Figure 2(b). The current states of the neurons are in the left layer. The weighted sum inputs are sent to the right layer, and the neurons of the right layer update. Subsequently, the states of neurons in the right layer are sent to the left layer.

### 2.2. Projection Rule

Learning is defined as determining the connection weights that make the training patterns fixed. The projection rule is one of the learning algorithms. We denote the *p*th training pattern by **x**^*p*^ = (*x*_1_^*p*^, *x*_2_^*p*^,…, *x*_*N*_^*p*^)^*T*^, where the superscript *T* refers to the transpose matrix. Let *P* be the number of training patterns. We define the training matrix *X* as follows:

(3) $$\begin{array}{c} X=\left(\begin{array}{cccc} x_{1}^{1} & x_{1}^{2} & \cdots & x_{1}^{P}\\ x_{2}^{1} & x_{2}^{2} & \cdots & x_{2}^{P}\\ \vdots & \vdots & \ddots & \vdots \\ x_{N}^{1} & x_{N}^{2} & \cdots & x_{N}^{P} \end{array}\right). \end{array}$$

By the projection rule, the connection matrix is determined as *W* = *X*(*X*^*∗*^*X*)^−1^*X*^*∗*^, where the superscript *∗* denotes the Hermitian conjugate matrix. This connection matrix is Hermitian and fixes the vector **x**^*p*^. For any integer *k*, the vector *e*^2*kiθ*_*K*_^**x**^*p*^ is referred to as the rotated pattern of **x**^*p*^. The rotated patterns of the training patterns are also fixed.

### 2.3. Recall Algorithm

The recall algorithm proposed by Lee is described. We utilize the synchronous mode for recall. A CHNN converges to a fixed point or a cycle. Fixed points are divided into two categories, local and global minima. We can determine whether a fixed point is a local or global minimum. In a fixed point, we calculate the weighted sum inputs. If all the lengths of the weighted sum inputs are 1, the fixed point is a global minimum. Otherwise, it is a local minimum.

Lee proposed exiting a local minimum or a cycle by changing a neuron's state. In this work, we added a small noise to a fixed vector in order to exit a local minimum or a cycle.

### 2.4. Fast Recall Algorithm

We only modify the weighted sum input for fast recall algorithm. The modified weighted sum input ${\tilde{I}}_{j}$ is as follows:

(4) $$\begin{array}{c} {\tilde{I}}_{j}=\sum_{k\neq j}w_{jk}x_{k}. \end{array}$$

This modified weighted sum input has often been utilized in the asynchronous mode. When the CHNN is trapped at a fixed point, we can determine whether the fixed point is a local or global minimum by checking $I_{j}={\tilde{I}}_{j}+w_{jj}x_{j}$.

## 3. Computer Simulations

We performed computer simulations to compare two recall algorithms. The number of neurons was *N* = 100. After a training pattern with noise was given to the CHNNs, the retrieval of the original training pattern was attempted. The noise was added by replacing each neuron's state with a new state at the rate of *r*, referred to as the Noise Rate. The new state was randomly selected from *K* states. It was allowed for the same state as the previous state to be selected.

Here we describe the recall process in the computer simulation.

1. A training pattern with noise was given to the CHNN.
2. The CHNN continued to update in the synchronous mode until it reached a fixed point or a cycle.
3. If the CHNN was trapped at a local minimum or a cycle, the noise was added at the rate 0.05 and the procedure returned to 2. Otherwise, the recall process was terminated.

After updating, if the pattern was identical to the pattern that preceded the previous pattern, the CHNN was trapped at a cycle. If the pattern is equal to the previous pattern, the CHNN was trapped at a local or global minimum. If the CHNN did not achieve a global minimum before 10,000 iterations, the recall process was terminated. We generated 100 training pattern sets randomly. For each training pattern, we performed 100 trials. Therefore, the number of trials was 10,000 for each condition.

**Figure 3:**
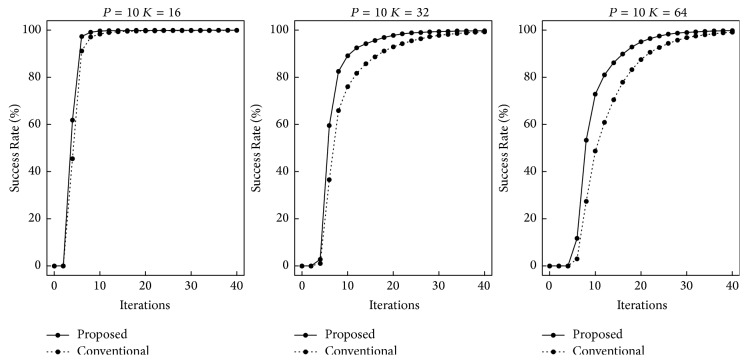
Recall speed in the case of *P* = 10.

**Figure 4:**
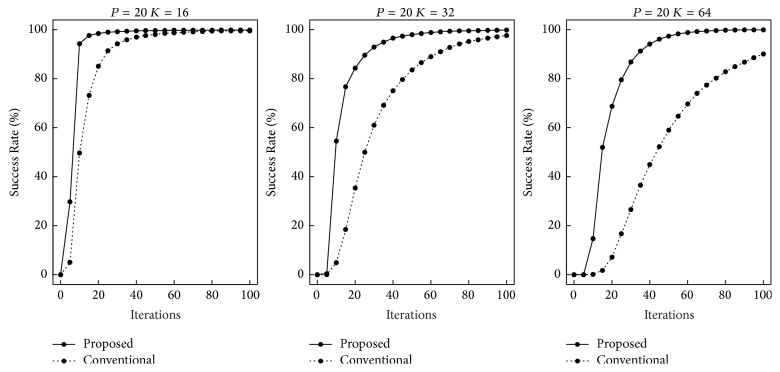
Recall speed in the case of *P* = 20.

**Figure 5:**
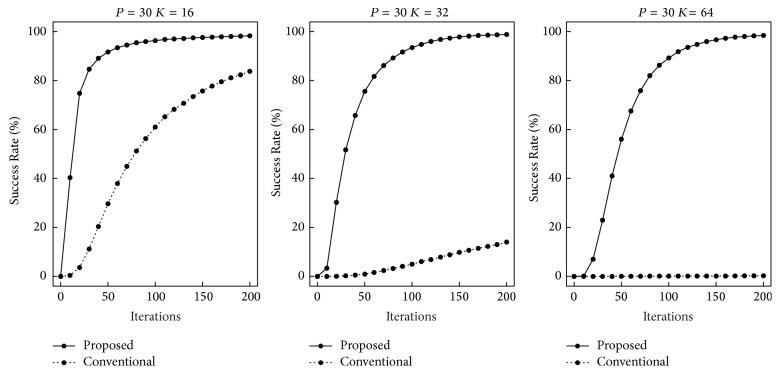
Recall speed in the case of *P* = 30.

First, we performed computer simulations for the recall speed. The numbers of training patterns used were *P* = 10,20, and 30. The quantization levels were *K* = 16,32, and 64. If a trial achieved a global minimum, it was regarded as successful. We randomly selected a training pattern and added noise at the rate of 0.3 in each trial. Figures 3–5 show the rates of trials that reached global minima by the indicated iterations. The horizontal and vertical axes indicate the iterations and the Success Rate, respectively. The ranges of iterations are different in each figure. As *K* and *P* increased, more iterations were required. In the case of *P* = 30 and *K* = 64, most trials did not achieve global minima until 10,000 iterations. In all the cases, our proposed recall algorithm was faster. As *P* and *K* increased, the differences became larger.

**Figure 6:**
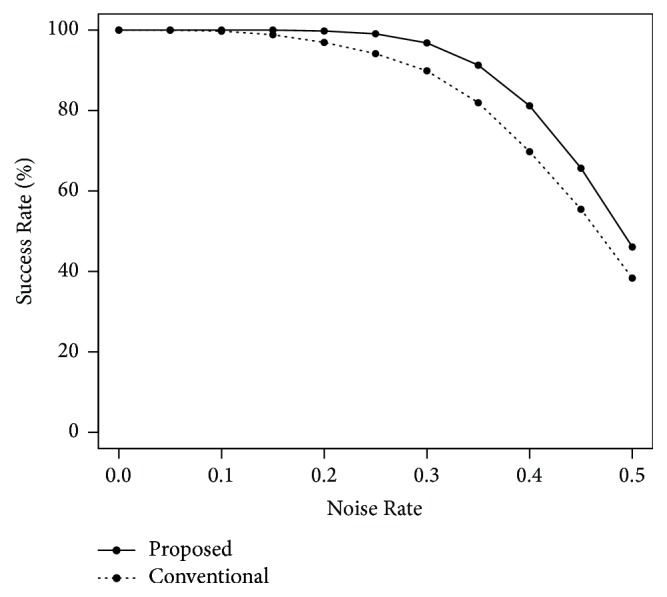
Noise tolerance in the case of *P* = 30 and *K* = 16.

**Figure 7:**
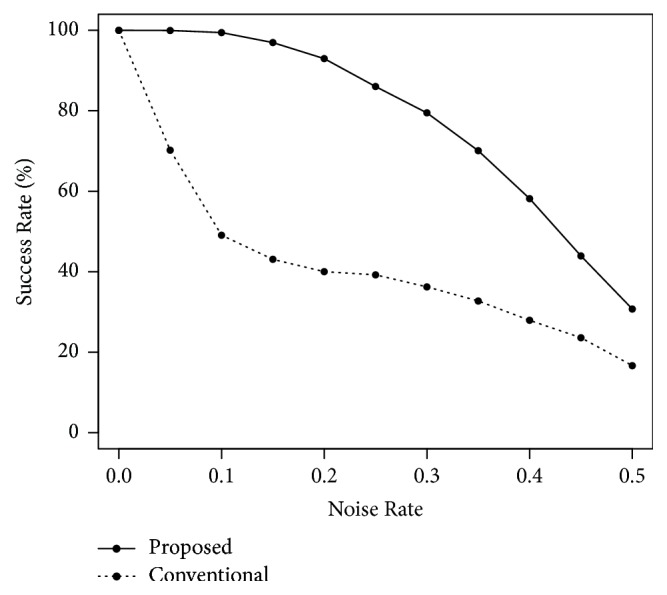
Noise tolerance in the case of *P* = 30 and *K* = 32.

Next, we performed computer simulations for the noise tolerance. The number of training patterns was *P* = 30, and the quantization levels were *K* = 16 and 32. We carried out the simulations using the training patterns with a Noise Rate of 0.3.  Figure 6 shows the results in the case of *K* = 16. The horizontal and vertical axes indicate the Noise Rate and the Success Rate, respectively. The proposed recall algorithm slightly outperformed the conventional algorithm.  Figure 7 shows the result in the case of *K* = 32. The noise tolerance of the conventional algorithm was very low. That of our proposed algorithm highly exceeded the conventional algorithm.

## 4. Discussion

**Figure 8:**
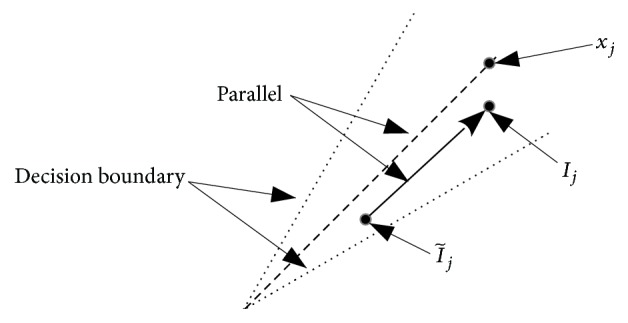
Autoconnections stabilize the states of neurons.

The computer simulations for the recall speed show that the conventional algorithm tended to be trapped at the local minima and the cycles. Autoconnections worked to stabilize the states of the neurons. When *w*_*jj*_*x*_*j*_ is added to ${\tilde{I}}_{j}$, the weighted sum input ${\tilde{I}}_{j}$ moves parallel to the line through 0 and *x*_*j*_ (Figure 8). Then, *I*_*j*_ is located far from the decision boundary. Some unfixed points would be stabilized to become fixed points. Thus, the autoconnections generate many fixed points and the CHNNs are easily trapped.

In the case of *P* = 30 and *K* = 32, most of the trials achieved global minima by 10,000 iterations. Therefore, many trials were trapped at the rotated patterns in Figure 7. During the repetition to exit the local minima and the cycles, the states of the CHNN would be far from the training pattern and would finally reach a rotated pattern. In the case of *P* = 30 and *K* = 64, most trials never achieved global minima until 10,000 iterations. With a too frequent addition of noise, the searching would become almost a random walk, and the trials could not achieve a global minimum.

## 5. Conclusion

Lee improved the noise tolerance of CHNNs with the projection rule by detecting and exiting the local minima and the cycles. We proposed a new recall algorithm to accelerate the recall. Our proposed recall algorithm eliminates the local minima and the cycles and accelerated the recall. In addition, our proposed recall algorithm improved the noise tolerance. On the other hand, the conventional recall algorithm hardly completes the recall in cases in which *K* and *P* are large. The local minima and the cycles are obviously the results of incorrect recall. Getting out of them certainly improves the noise tolerance, though it takes long time to complete the recall. The recall algorithms should be studied so as to reduce falling further into the local minima and the cycles.
